# Trait aggression is associated with five‐factor personality traits in males

**DOI:** 10.1002/brb3.2175

**Published:** 2021-05-25

**Authors:** Vibeke H. Dam, Liv Vadskjær Hjordt, Sofi da Cunha‐Bang, Dorte Sestoft, Gitte Moos Knudsen, Dea Siggaard Stenbæk

**Affiliations:** ^1^ Neurobiology Research Unit The Neuroscience Centre Copenhagen University Hospital Rigshospitalet Copenhagen Denmark; ^2^ Faculty of Health and Medical Sciences University of Copenhagen Copenhagen Denmark; ^3^ Clinic of Forensic Psychiatry Ministry of Justice Copenhagen Denmark

**Keywords:** aggression, Buss–Perry Aggression Questionnaire, five‐factor personality, violent offenders

## Abstract

**Background:**

Although aggression is conceptualized as a dimensional construct with violent behavior representing the extreme end of a spectrum, studies on the involvement of personality traits in human aggression have typically only included data representing a restricted spectrum of aggressive behaviors.

**Methods:**

In the current study, we therefore examine whether trait aggression is associated with five‐factor model personality traits in an enriched sample of 259 men with a broad continuum of trait aggression, ranging from very low to very high including 39 incarcerated aggressive violent offenders. All participants completed the NEO Personality Inventory‐Revised (NEO PI‐R) and the Buss–Perry Aggression Questionnaire (BPAQ). The association between each of the five NEO PI‐R personality traits and trait aggression (BPAQ) was investigated using five linear regression models, covarying for group status, age and educational level.

**Results:**

Higher BPAQ scores were positively associated with Neuroticism and negatively associated with Agreeableness and Conscientiousness.

**Conclusion:**

Our results indicate that those high in Neuroticism and low in Agreeableness and Conscientiousness are at higher risk of exhibiting aggressive behavior, underlining the relevance of these higher order personality traits in understanding aggressive behavior. We argue that studying individual personality differences should be offered a greater attention within violent and criminal behaviors.

## Graphical abstract

In this study, we for the first time examine whether trait aggression is associated with five‐factor model personality traits in an enriched sample of 259 men with a broad continuum of trait aggression, ranging from very low to very high including 39 incarcerated aggressive violent offenders. Our results indicate that individuals with high scores on Neuroticism and low scores on Agreeableness and Conscientiousness are at higher risk of exhibiting aggressive behavior, and underline the relevance of these higher order personality traits in understanding aggressive behavior.

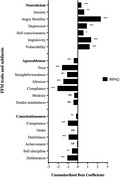

## 1 INTRODUCTION

Aggression and interpersonal violence constitute a major societal burden with far‐reaching negative consequences for both victims and perpetrators. Accordingly, improvements of preventative measures, better risk assessment, and targeted rehabilitative programs for violent offenders are a global priority (Krug et al., 2002; WHO, United Nations Office on, Crime, & United Nations Development, 2014). Studies have identified individual personality differences as an important factor in aggressive and antisocial behavior (McMurran, 2009). For instance, pathological personality constructs such as borderline and antisocial personality disorder (Mancke et al., 2018; Yu et al., 2012), psychopathy (Blais et al., 2014), and narcissism (Lambe et al., 2018) constitute strong risk factors in aggressive and antisocial behavior. However, as the criteria for several of these constructs—in particular antisocial personality disorder and psychopathy—are based on a history of aggressive and antisocial behavior, this creates a circular logic. Nonpathological personality constructs, for example, five‐factor model (FFM) personality and Cloninger's Temperament and Character model, have also been associated with aggression, but have typically received less attention within the literature (Miller et al., 2001). Personality traits are defined as psychological and behavioral characteristics that remain stable over time and across different contexts (Costa & McCrae, 2005). A major index of personality functioning is the FFM which describes five broad personality traits: Neuroticism, Extraversion, Openness to Experience, Agreeableness, and Conscientiousness, with each trait consisting of six distinct subfacets. The FFM has gained wide usage due to converging evidence of its discriminant and consensual validity, stability, heritability, cross‐cultural invariance, and predictive utility (Costa & McCrae, 2005).

A large body of studies support the involvement of FFM personality traits in human aggression, with high Neuroticism, low Agreeableness, and low Conscientiousness exhibiting the strongest association with aggressive and antisocial behavior [for meta‐analyses see Jones et al. (2011) and Miller and Lynam (2001)]. Although aggression is perceived as a dimensional construct where violent behavior represents the extreme end of the aggression spectrum, studies regarding the involvement of personality traits in human aggression have typically either included university students and inmates convicted of nonviolent or violent crimes and hence only included data from a restricted spectrum of aggressive behaviors. For example, Hansen et al. (2011) reported that inmates convicted of violent crimes exhibited lower scores of Agreeableness compared to inmates convicted of nonviolent crimes while Mouilso and Calhoun (2012) found that nonconvicted student sexual offenders had lower levels of Agreeableness and Conscientiousness than nonoffender students. Barlett and Anderson (2012) reported that high Neuroticism, low Agreeableness, and low Conscientiousness were linked to increases in aggressive emotions and physical aggression in a large sample of university students, mirrored by the findings of Klimstra et al. (2010) in adolescents. For evaluation of how personality traits are related to aggression as a dimensional construct, studies should include a cohort representing a broad continuum of aggression levels ranging from low‐aggressive individuals to proven aggressive individuals. However, to our knowledge no such study has been carried out. In addition, as emphasized by Jones et al. (2011), future studies should examine the involvement of FFM personality traits in human aggression at subfacet level, as this provides information on the broadest possible dimensions of personality and appear to have a greater predictive ability than the overarching personality five traits (see for example Vedel et al., 2015).

In this study, we therefore integrate FFM personality data in an enriched cohort of men with a broad continuum of trait aggression, ranging from very low to very high including 39 incarcerated aggressive violent offenders, to evaluate whether personality is associated with levels of trait aggression (indexed as total Buss–Perry Aggression Questionnaire [BPAQ] score). Based on the existing literature, we hypothesize that Neuroticism scores are positively correlated with BPAQ scores and Agreeableness and Conscientiousness scores are negatively correlated with BPAQ scores.

## 2 MATERIALS AND METHODS

### 2.1 Participants

The study consisted of 259 male participants (Age: 29.1 ± 8.3, mean ± *SD*). To ensure that the study cohort represented a wide spectrum of trait aggression beyond that which were reported by a sample of 183 healthy male volunteers from our established Center for Integrated Molecular Brain Imaging (Cimbi) database (Knudsen et al., 2016), two subgroups at each tail of the aggression spectrum were additionally included; one subgroup included 39 offenders with a documented history of violent crimes (e.g., murder, attempted murder, rape, aggravated assault) and a second subgroup included 37 healthy nonoffender individuals with low levels of trait aggression.

The violent offenders were recruited from two high‐security state prisons in Denmark; 43 offenders were initially included in the study, however, one inmate was moved to another prison before completing the study program, one inmate was missing essential questionnaire data, and two inmates did not fit the profile of a violent offender upon closer review^1^ and were therefore excluded before data analysis was commenced. Individuals from the community sample and the healthy nonoffenders with low levels of trait aggression were recruited from the community via online advertisements and flyers posted at work places and trade schools in the greater Copenhagen area. Specific for the healthy nonoffenders, they were matched to the violent offenders on age and sex and all had low self‐reported levels of aggression (BPAQ scores ≤65) and presented with no criminal record. We selected all participants according to the following criteria: Absence of past or present major depressive disorder, bipolar disorder, schizophrenia, absence of significant somatic illness or brain trauma, no current use of drugs, alcohol, or psychotropic medication, and nonfluency in Danish.

All individuals were recruited by advertisement for different research protocols approved by the Ethics Committee of Copenhagen and Frederiksberg, Denmark. After a complete description of the respective studies, written informed consent was obtained from all individuals. A thorough description of the Cimbi database has been published elsewhere (Knudsen et al., 2016).

### 2.2 Measures

#### 2.2.1 Trait personality: Revised NEO Personality Inventory

The Danish version of the NEO PI‐R is a self‐report questionnaire used to assess the construct of FFM personality and has been normed in a sample of 600 Danish individuals (300 males) (Skovdahl et al., 2011). It comprises five domains of personality: Neuroticism, Extraversion, Openness to Experience, Agreeableness, and Conscientiousness with each domain consisting of six subfacets. The questionnaire contains 240 items (e.g., from trait Neuroticism: “I am not a worrier” or “I often get angry at the way people treat me”) rated on a 5‐point Likert scale from 0 (strongly disagree) to 4 (strongly agree) (Costa & McCrae, 2005).

#### 2.2.2 Trait aggression: Buss–Perry Aggression Questionnaire

The Danish version of the BPAQ is a self‐report questionnaire assessing aggressive tendencies (Buss & Perry, 1992). BPAQ is a comprehensive, widely used and well‐validated measure of aggression (Buss & Perry, 1992; da Cunha‐Bang et al., 2013; Harris, 1997). It comprises four components of aggression: Verbal aggression, physical aggression, hostility, and anger. It contains 29 items (e.g., from the physical aggression subscale: “If I have to resort to violence to protect my rights, I will” and “I have become so mad that I have broken things”) rated on a 5‐point Likert scale from 1 (Extremely uncharacteristic of me) to 5 (Extremely characteristic of me).

### 2.3 Statistical analyses

Group differences in demographics, FFM personality traits and levels of trait aggression were evaluated with Mann–Whitney *U* tests. Internal consistency within FFM traits and BPAQ scores were examined with Cronbach's alpha (*α*).

To test our hypotheses, the associations between each of the five FFM personality traits and trait aggression (total BPAQ score) were evaluated separately using five linear regression models. The models were adjusted for group status (healthy nonoffenders, community sample, and violent offenders) and for variables known to be associated with personality or aggression, that is, age and educational level (Skovdahl et al., 2011). Whenever a FFM personality trait was significantly associated with trait aggression, we conducted follow‐up analyses to examine the associations between the constitutive subfacets of the trait and trait aggression. Bootstrapping was used to calculate 95% Bias‐Corrected accelerated Confidence Intervals (BCa CI) for the regression estimates.

Model assumptions were evaluated by visual inspection of raw score or model residuals. Outliers were defined as scores >3.29 *SD* from the mean. Alpha levels were set at 0.05 for statistical significance. *p*‐Values were adjusted by the Bonferroni–Holm multiple comparison procedure (Holm, 1979): *p*‐values in analyses on traits were adjusted for five tests, and *p*‐values in analyses on subfacets were adjusted for six tests within each trait. All other *p*‐values are reported unadjusted. All statistical analyses were carried out in SPSS (v.25.0).

## 3 RESULTS

Table 1 shows participants’ demographic characteristics, mean FFM trait and BPAQ scores and significant group differences in demographics, BPAQ, and FFM scores. Three outliers were detected for traits Neuroticism, Agreeableness, and Conscientiousness, respectively. As removal of the outliers did not significantly change the results, they were included in the final analyses.

**TABLE 1:** Descriptive data

	Total (*N* = 259)	Nonoffenders (*n* = 37)	Community sample (*n* = 183)	Violent offenders (*n* = 39)
	Mean ± *SD* (range)	Mean ± *SD* (range)	Mean ± *SD* (range)	Mean ± *SD* (range)
Age in years^a^, ^b^	29.1 ± 8.3 (18–59)	33.6 ± 10.3 (20–55)	27.4 ± 6.8 (18–59)	32.8 ± 9.9 (19–59)
Education in years^a^, ^b^, ^c^	11.3 ± 1.5 (0–12)	11.3 ± 1.2 (8–12)	11.8 ± 0.7 (7–12)	9.1 ± 2.4 (0–12)
Total BPAQ score^a^, ^b^, ^c^	63.4 ± 19.1 (32–129)	50.9 ± 8.2 (32–66)	61.1 ± 15.6 (33–116)	86.3 ± 23.0 (43–129)
Neuroticism^b^, ^c^	73.3 ± 21.4 (17–128)	65.7 ± 19.3 (17–107)	72.7 ± 20.4 (24–128)	83.3 ± 24.7 (20–128)
Extraversion^c^	118.2 ± 18.9 (53–157)	114.3 ± 20.2 (62–152)	120.3 ± 18.6 (53–157)	111.9 ± 17.2 (73–145)
Opennes to Experience^b^, ^c^	116.5 ± 19.3 (68–165)	118.3 ± 22.1 (76–165)	119.2 ± 18.4 (68–163)	101.9 ± 14.1 (79–139)
Aggreableness^a^, ^b^	118.3 ± 18.3 (47–168)	132.1 ± 15.9 (101–168)	116.8 ± 17.3 (47–168)	112.1 ± 19.0 (84–161)
Conscientiousness	113.5 ± 21.1 (42–162)	113.4 ± 19.0 (80–159)	112.8 ± 21.6 (42‐162)	116.6 ± 20.8 (77–158)

Note

Participants’ demographic characteristics and mean raw FFM trait and BPAQ scores. Education score is measured with the Online Stimulant and Family History Assessment Module. Differences between groups in demographics, FFM personality traits and BPAQ scores were evaluated with Mann–Whitney *U* tests.

Abbreviation: *SD*, Standard deviation.

^a^ Nonoffenders versus community sample, *p* < .05.

^b^ Nonoffenders versus community sample, *p* < .05.

^c^ Community sample versus violent offenders, *p* < .05.

### 3.1 Internal consistency

Internal reliability indexed with Cronbach's *α* ranged from good to excellent for BPAQ total score and all FFM personality traits: BPAQ (α = 0.92), Neuroticism (*α* = 0.91), Extraversion (*α* = 0.87), Openness to Experience (*α* = 0.87), Agreeableness (*α* = 0.88), and Conscientiousness (*α = *0.91).

### 3.2 FFM personality traits and trait aggression

**FIGURE 1:**
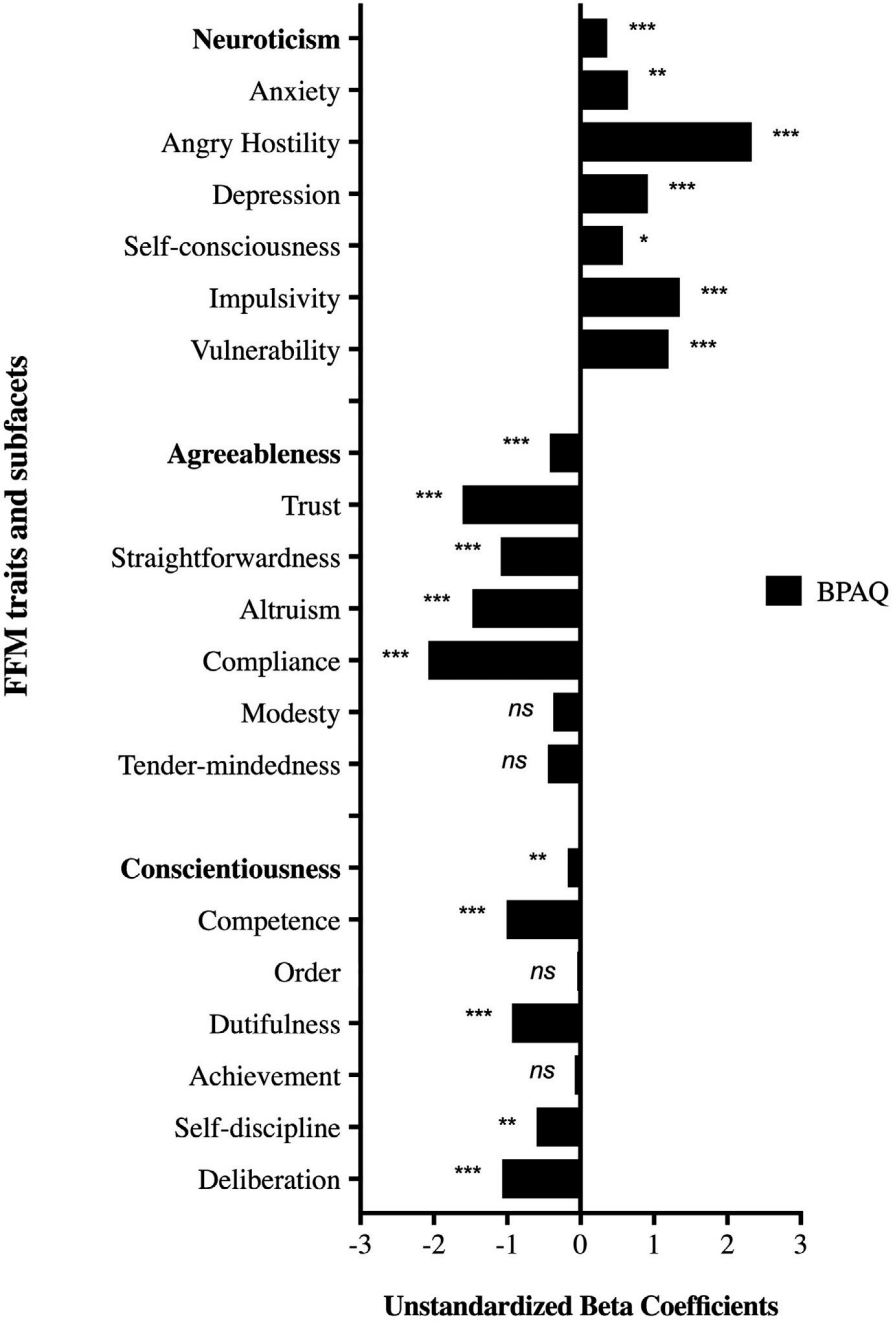
Association between trait aggression and FFM personality traits

Figure 1 shows unstandardized beta coefficients of the linear regression models evaluating the association between total BPAQ scores and Neuroticism, Agreeableness and Conscientiousness. Associations between total BPAQ and each of the five FFM personality traits, as well as subfacets for significant traits, are presented in Table 2. Associations between each of the five FFM personality traits and BPAQ subfacets are listed in Table S1.

**TABLE 2:** Associations between FFM personality traits, as well as subfacets for significant traits and BPAQ

	Unstandardized Beta coefficients	*SE*	BCa 95% CI – lower	BCa 95% CI – higher	*p*‐Values Bonferroni adjusted^a^
Neuroticism	0.36	0.04	0.28	0.45	<.001
Anxiety	0.64	0.20	0.28	1.03	.007
Angry Hostility	2.33	0.15	2.05	2.60	<.001
Depression	0.92	0.17	0.59	1.25	<.001
Self‐consciousness	0.57	0.21	0.16	1.00	.040
Impulsivity	1.35	0.21	0.96	1.73	<.001
Vulnerability	1.20	0.26	0.70	1.72	<.001
Extraversion	−0.06	0.06	−0.17	0.06	1.000
Openness to Experience	−0.01	0.06	−0.12	0.11	1.000
Agreeableness	−0.42	0.05	−0.51	−0.31	<.001
Trust	−1.61	0.20	−2.03	−1.19	<.001
Straightforwardness	−1.09	0.18	−1.46	−0.72	<.001
Altruism	−1.47	0.29	−2.03	−0.89	<.001
Compliance	−2.08	0.22	−2.50	−1.64	<.001
Modesty	−0.37	0.20	−0.79	0.03	.412
Tender‐mindedness	−0.45	0.26	−0.94	0.10	.503
Conscientiousness	−0.18	0.05	−0.28	−0.08	<.01
Competence	−1.01	0.27	−1.57	−0.46	.001
Order	−0.03	0.23	−0.48	0.43	1.000
Dutifulness	−0.93	0.23	−1.40	−0.48	<.001
Achievement	−0.08	0.19	−0.47	0.29	1.000
Self‐discipline	−0.60	0.18	−0.96	−0.25	<.01
Deliberation	−1.07	0.22	−1.51	−0.61	<.001

Notes

Unstandardized beta coefficients denote the point estimate of the linear regression model for a given effect.

Abbreviations: BCa CL, Bias‐Corrected accelerated Confidence Intervals; *SE*, standard error.

^a^ *p*‐Values were adjusted by the Bonferroni multiple comparison procedure (Holm, 1979): *p*‐values in analyses on traits were adjusted for five tests, and *p*‐values in analyses on subfacets were adjusted for six tests within each trait.

*Neuroticism* scores were positively correlated with BPAQ scores and this association was observed for all Neuroticism subfacets; Anxiety, Angry Hostility, Depression, Self‐consciousness, Impulsivity, and Vulnerability. *Agreeableness* scores were negatively correlated with BPAQ scores and this association was also observed for the Agreeableness subfacets Trust, Straightforwardness, Altruism, and Compliance. We did not observe a significant effect for the subfacets Modesty and Tender‐mindedness. *Conscientiousness* scores were negatively correlated with BPAQ scores and this association was also observed for the Conscientiousness subfacets Competence, Dutifulness, Self‐discipline, and Deliberation. We did not observe a significant effect for the subfacet Order. *Extraversion* and *Openness to Experience* scores were not associated with BPAQ scores.

## 4 DISCUSSION

The aim of this study was to examine the relationship between levels of trait aggression as a dimensional construct and traits from the FFM of personality. To our knowledge, this is the first study to use an enriched cohort by including individuals representing the full spectrum of aggression levels. We show that trait aggression is positively associated with Neuroticism and negatively associated with Agreeableness and Conscientiousness, replicating the reported findings from a meta‐analysis study by Jones et al. (2011). Our results imply that those high in Neuroticism and low in Agreeableness and Conscientiousness are at higher risk of exhibiting aggressive behavior.

Neuroticism describes the overall tendency to experience negative emotions (Costa & McCrae, 2005), including higher stress reactivity, increased feelings of hostility and anger, poor impulse control, and increased sensitivity to frustration and provocations (Bettencourt et al., 2006; Zajenkowska et al., 2013). The use of the narrower subfacets allowed for a parsing of the trait level findings. While all six Neuroticism subfacets demonstrated significant associations with trait aggression, the facets of Angry Hostility and Impulsivity presented with the strongest relationship, indicating that the propensity to feel angry hostile emotions and poor impulse control are more fundamental to aggressive behaviors than other Neuroticism subfacets (e.g., Anxiety and Self‐consciousness). However, this observation may be partly explained by significant predictor‐criterion overlap in the questionnaires used, particularly for the two BPAQ subscales designed to assess traits related to anger and hostility. Although FFM is a result of basic personality research and do not explicitly reference aggressive acts, some items on the NEO and BPAQ questionnaire are close to identical: For example, “I am perceived as fiery and temperamental” from Neuroticism subfacet Angry Hostility and “Some of my friends think I am a hothead” from BPAQ subscale Anger. Thus, it is not surprising that the Angry Hostility subfacet of the NEO PI‐R is such a strong correlate to BPAQ.

The central role of Agreeableness in aggression is well documented (Jones et al., 2011;. Miller & Lynam, 2001) and our findings align with previous results. While the narrower subfacets analyses showed that four of six Agreeableness facets demonstrated significant negative relation to trait aggression, the facets of Trust, Altruism and Compliance, presented with the strongest negative relationship. According to Costa and McCrae (2005), high levels of Agreeableness promote prosocial behaviors such as cooperativeness, kindness, and altruism, while low Agreeableness promote the tendency to feel less sympathy and empathy toward others (Graziano et al., 2007). Thus, low Agreeableness may lead to an increase in interpersonal conflict through the disinhibition of social and relational regulatory mechanisms mediated by lack of empathic attunement which allows the individual to more easily act on their aggressive and violent impulses (Bettencourt et al., 2006). Interestingly, evidence suggest that the presence of high Neuroticism may not be sufficient to promote aggressive and violent behavior on its own but instead must be in conjunction with low Agreeableness (Ode et al., 2008). Thus, one way to interpret the interaction between Neuroticism and Agreeableness is that the negative bias and emotional dysregulation indexed by high Neuroticism makes the individual more sensitive to situational triggers such as provocations or perceived insults which in combination with low Agreeableness, may facilitate hostile and aggressive behavior. This could in turn contribute to the negative effect on mental health associated with high Neuroticism, as repeated antagonistic and confrontational interactions with others might enforce the tendency to interpret the world and the motivations of others negatively (Costa & McCrae, 2005).

Conscientiousness describes the propensity to be deliberate, goal‐oriented, and disciplined (Costa & McCrae, 2005). Within the literature, a small but consistent negative association has been reported (Jones et al., 2011), matching our findings in the present study. The narrower subfacets analyses showed that four of six Conscientiousness facets demonstrated small but statistically significant negative relation to trait aggression: Deliberation, Self‐discipline, Dutifulness, and Competence. The link between Conscientiousness and aggression is less clear than those of Neuroticism and Agreeableness, but one interpretation may be that individuals low on Conscientiousness are more impulsive and focus less on the potential consequences of their actions and thus are less deterred by the negative social consequences of aggressive and disruptive behaviors.

While FFM personality traits do not translate directly into behaviors, they are thought to form the basis on which the individual develops manifest psychological characteristics such as beliefs, values, and attitudes (Costa & McCrae, 2005). These characteristics further take form through interaction with the immediate environment and the accumulation of experience. Thus, based on our findings, it can be hypothesized that personality characteristics that are aligned with low scores on Neuroticism and high scores on Agreeableness and Conscientiousness are protective against violent offending. More specifically, this would include traits such as emotional stability, inhibition of impulsive behaviors, and a general tendency toward cooperativeness with others. Psychosocial interventions that aim to reduce violent offending should thus focus on interventions such as learning conflict‐resolution techniques, trust training, learning to regulate negative emotional states, and learning strategies to identify and control impulsive behaviors. Future studies should examine the moderation of personality differences on outcomes of psychotherapy and other psychosocial interventions commonly used to treat aggressive behavior in community.

### 4.1 Methodological considerations

A notable strength of the present study is the inclusion of a large and enriched cohort of men with varying degree of aggressive traits, which allowed for detection of relations between FFM personality traits and trait aggression along the aggression spectrum. In addition, the use of Bonferroni–Holm correction for multiple comparisons in analyses on domains and subfacets lowers the risk of false‐positive findings. Moreover, reporting results on subfacet level provides information on the broadest possible dimensions of personality and could be hypothesis‐generating for future studies on self‐perceived personality characteristics in individuals exhibiting different levels of trait aggression.

However, the results should be interpreted in light of some important limitations. First, the present study was based on self‐report measures which has been criticized for not always accurately reflecting real‐world behavioral patterns (Baumeister, Vohs, & Funder, 2007) and being vulnerable to response biases (Domino & Domino, 2006; Lundmann & Villadsen, 2016). While studies based on self‐reports and spouse ratings on the NEO PI‐R questionnaire support the validity of the personality traits measured (Costa & McCrae, 2005), it has been reported that psychopathic traits such as Machiavellian egocentricity and externalizing blame are associated with greater success in faking a positive personality profile (MacNeil & Holden, 2006). This may be particularly relevant as 80% of the violent offenders met the criteria for psychopathy based on the Hare Psychopathy Checklist‐Revised (PCL‐R) when using a cutoff score of ≥25 (Brazil & Forth, 2016). In addition, the study sample consisted of only men, which means we cannot generalize the results to women.

## CONFLICT OF INTEREST

No authors have any conflict of interest and no financial disclosures.

### PEER REVIEW

The peer review history for this article is available at https://publons.com/publon/10.1002/brb3.2175.

## Supporting information

Table S1

## Data Availability

The data that support the findings of this study are available on request from the corresponding author. The data are not publicly available due to privacy or ethical restrictions

## Footnotes

^1^
A thorough review of their criminal records showed that the crimes the offenders had been convicted of did not involve direct acts of aggression or violence.
