# Supplementary material for: Trait aggression is associated with five‐factor personality traits in males
Source: Brain Behav. 2021 May 25;11(7):e02175. doi: 10.1002/brb3.2175 (PMC8323029; doi:10.1002/brb3.2175)
Supplement: Supplementary file 1 — Table S1 [file BRB3-11-e02175-s001.docx]

|  |  |  |  |  |  |
| --- | --- | --- | --- | --- | --- |
|  |  |  |  |  |  |
|  |  |  |  |  |  |
|  |  |  |  |  |  |
|  |  |  |  |  |  |
|  |  |  |  |  |  |
|  |  |  |  |  |  |
|  |  |  |  |  |  |
|  |  |  |  |  |  |
|  |  |  |  |  |  |
|  |  |  |  |  |  |
|  | | | | | |

| **Table S1. Association between FFM personality traits and BPAQ subfacets** | | | | | | |  |  |  |  |  |  |
| --- | --- | --- | --- | --- | --- | --- | --- | --- | --- | --- | --- | --- |
|  |  | Physical aggression | |  | Verbal aggression | |  | Anger | |  | Hostility | |
|  |  | *Unstandardized Beta coefficients* | *P-values Bonferroni adjusted^a^* |  | *Unstandardized Beta coefficients* | *P-values Bonferroni adjusted^a^* |  | *Unstandardized Beta coefficients* | *P-values Bonferroni adjusted^a^* |  | *Unstandardized Beta coefficients* | *P-values Bonferroni adjusted^a^* |
| Neuroticism |  | 0.09 | <0.001 |  | 0.02 | 0.203 |  | 0.11 | <0.001 |  | 0.15 | <0.001 |
| Extraversion |  | -0.004 | 1.000 |  | 0.03 | 0.160 |  | -0.01 | 1.000 |  | -0.05 | 0.031 |
| Openness to Experience |  | -0.03 | 0.948 |  | 0.02 | 0.242 |  | 0.004 | 1.000 |  | 0.004 | 1.000 |
| Agreeableness |  | -0.15 | <0.001 |  | -0.10 | <0.001 |  | -0.11 | <0.001 |  | -0.09 | <0.001 |
| Conscientiousness |  | -0.06 | 0.005 |  | 0.004 | 1.000 |  | -0.04 | 0.055 |  | -0.06 | 0.002 |
| Notes: Unstandardized beta coefficients denotes the point estimate of the linear regression model for a given effect. Covariates include age, education and group as co-variates.  ^a^ P-values were adjusted by the Bonferroni-Holm multiple comparison procedure (Holm, 1979): P-values in analyses on traits were adjusted for four tests, and p-values in analyses on sub-facets were adjusted for six tests within each trait. N = 259 | | | | | | | | | | | | |
